# Application of individualized multimodal radiotherapy combined with immunotherapy in metastatic tumors

**DOI:** 10.3389/fimmu.2022.1106644

**Published:** 2023-01-12

**Authors:** Xiaoqin Ji, Wanrong Jiang, Jiasheng Wang, Bin Zhou, Wei Ding, Shuling Liu, Hua Huang, Guanhua Chen, Xiangdong Sun

**Affiliations:** Department of Radiation Oncology, Affiliated Jinling Hospital, Medical School of Nanjing University, Nanjing, China

**Keywords:** cancer, metastatic tumor, high-dose radiotherapy, low-dose radiotherapy, immunotherapy

## Abstract

Radiotherapy is one of the mainstays of cancer treatment. More than half of cancer patients receive radiation therapy. In addition to the well-known direct tumoricidal effect, radiotherapy has immunomodulatory properties. When combined with immunotherapy, radiotherapy, especially high-dose radiotherapy (HDRT), exert superior systemic effects on distal and unirradiated tumors, which is called abscopal effect. However, these effects are not always effective for cancer patients. Therefore, many studies have focused on exploring the optimized radiotherapy regimens to further enhance the antitumor immunity of HDRT and reduce its immunosuppressive effect. Several studies have shown that low-dose radiotherapy (LDRT) can effectively reprogram the tumor microenvironment, thereby potentially overcoming the immunosuppressive stroma induced by HDRT. However, bridging the gap between preclinical commitment and effective clinical delivery is challenging. In this review, we summarized the existing studies supporting the combined use of HDRT and LDRT to synergistically enhance antitumor immunity, and provided ideas for the individualized clinical application of multimodal radiotherapy (HDRT+LDRT) combined with immunotherapy.

## Introduction

Radiotherapy has long been the cornerstone of cancer treatment for curative purposes as well as palliative relief of symptoms, thereby improving quality of life (1–4). In addition to inducing irreparable DNA damage with direct cytotoxic effects on cancer cells, there is growing evidence that radiotherapy can modulate the immune system, resulting in systemic antitumor immunity (5, 6). This systemic response is called abscopal effect, that is, radiation targeting one tumor lesion can induce *in situ* vaccination by killing tumor cells, and then lead to the regression of distant unirradiated tumors (7–9). Although radiotherapy alone rarely induces abscopal effects, the potential systemic antitumor ability provides a good basis for radiotherapy combined with immunotherapy.

Immunotherapy has been recognized as an effective oncologic therapy. In particular, immune checkpoint inhibitors (ICIs) have achieved surprising clinical efficacy in the treatment of advanced solid tumors (10–12). However, only a minority of patients with advanced cancers can experience persistent and stable benefit from ICIs alone (13). As a result, many clinical trials are exploring the synergistic effect of radiation therapy combined with immunotherapy to enhance antitumor immunity (14–18). The updated data of PACIFIC Trial demonstrated robust and sustained overall survival (OS) and durable progression free survival (PFS) benefit with durvalumab after chemoradiotherapy in patients with unresectable, stage III non-small-cell lung cancer (NSCLC) (19). These compelling clinical evidences provide a basis for further exploration of the best combination regimen.

The stereotactic body radiation therapy (SBRT, also known as stereotactic ablative radiotherapy [SABR]) is increasingly used to deliver highly targeted high doses with fewer fractions, because of the high rates of local tumor control with tolerable toxicity (20–22). In addition, high-dose radiotherapy (HDRT, such as SBRT) is more immunogenic than conventional radiotherapy. HDRT can mobilize innate and adaptive immunity against tumors (23–26). Therefore, scholars focused on the combination of HDRT with immunotherapy to enhance the antitumor immunity of patients. Several clinical studies have shown that SBRT combined with ICIs can significantly improve the response rates in metastatic tumors with well tolerated (27–29). Despite previous progression on anti-PD-1 therapy, SBRT has reinvigorated a systemic response (30). Nevertheless, in some cases, SBRT in conjunction with ICIs may not eliminate distant tumors, and benefit only a small fraction of patients (31, 32). HDRT can sometimes have inhibitory effects on antitumor immunity, such as recruiting immunosuppressive cells and increasing the secretion of immunoregulatory cytokines (33, 34). It is urgent to overcome the immune-suppressive barriers to increase the beneficiary population of immunotherapy.

Many studies have shown that low-dose radiotherapy (LDRT), i.e., lower than 2 Gy/fraction, can effectively reprogram the stroma from an immunosuppressive to immunostimulatory and synergize with immunotherapy (35–37). Interestingly, the mechanisms by which HDRT and LDRT regulate the antitumor immune system appear to be complementary (38). Therefore, the use of the multimodal radiotherapy regimen, such as HDRT and LDRT, can achieve optimal antitumor effects. We first proposed the concept of multimodal radiotherapy, that is, the combination of different radiotherapy modalities, such as SBRT combined with intensity modulated radiotherapy (IMRT), SBRT combined with LDRT, HDRT combined with LDRT, etc. Savage et al., compared a single-dose ablative fractionation of 24Gy with 22Gy followed by 4 fractions of 0.5Gy targeting the local tumor in C57BL/6 mice. They found that the addition of LDRT delayed local tumor progression and significantly improved survival. In addition, survival was significantly increased after whole-lung radiated by low dose (0.5Gyx4f), 12 days after completion of the primary tumor radiation (20Gyx3f) (39). Furthermore, some preclinical and clinical studies showed that the multimodal radiotherapy (HDRT and LDRT) combined with immunotherapy can enhance systemic anti-tumor immune responses (40–44). However, there are many problems about this novel combination therapy strategy. For example, the selection of immunotherapy agents, the sequence of multimodal radiotherapy combined with immunotherapy, the dose of radiation, the number of fractions, the site of high-dose irradiation, and the site of low-dose irradiation.

In this review, we discussed the optimal radiotherapy regimens for enhancing antitumor immunity. First, we investigated the modulation of radiation on the immune system, including immunoenhancing and immunosuppressive effects. Furthermore, we described the different mechanisms of HDRT and LDRT in immune regulation. Finally, we studied the rationale for combining multimodal radiotherapy (HDRT and LDRT) with immunotherapy to enhance antitumor immune responses.

## The direct killing effect of radiation on tumor cells

Radiation therapy has been widely used to treat malignant tumors since the discovery of X-ray by Roentgen in 1895 (45). Approximately 60-70% of cancer patients require radiation therapy during treatment (7). In 1911, Regaud et al. proposed the concept of fractionated radiotherapy, in which a large doses can be divided into fractions over days or weeks (46). Nowadays, the conventional fractionated radiotherapy regimen is usually 1.8-2 Gy daily, 5 fractions/week. Hypofractionated radiotherapy refers to increasing the single irradiation dose >2 Gy, which has the advantage of shortening the treatment time span of patients and avoiding accelerated tumor proliferation after radiotherapy. Over the past few decades, the field of radiation has undergone tremendous technological innovations that can significantly reduce radiation damage to healthy tissues with modern radiotherapy techniques such as helical tomotherapy, IMRT, proton radiotherapy, SBRT and FLASH radiotherapy (22, 47–52).

Irradiation can directly cause DNA damage, such as single-strand breaks (SSBs), double-strand breaks (DSBs), DNA cross-links, and DNA-protein cross-links, resulting in therapeutic effects on tumor cells, such as apoptosis, necrosis, senescence, and mitotic abnormalities (53, 54). Irradiation can indirectly induce damage to DNA molecular chain in cancer cells by ionizing water molecules to generate H^+^ and OH^-^ (55). This indirect effect requires oxygen. Therefore, some hypoxic tumors are resistant to radiation, which is one of the reasons for tumor recurrence after radiotherapy. Hypoxia in hypoxic tumors causes less DNA damage than in well-oxygenated tumors at the same dose of radiation. In addition, hypoxia leads to activation of the hypoxia inducible factor (HIF) signaling pathway. Activation of HIF1 can affect the expression of hundreds of genes, including vascular endothelial growth factor (VEGF) and angiopoietin-1 (ANGPT1), which promote tumor survival (56). It also drives the expression of key enzymes in glycolysis, resulting in the accumulation of lactic acid, pyruvate, and the antioxidants glutathione and NADPH to limit DNA damage (57). Therefore, radiation alone is not enough to kill all cancer cells, and it is necessary to study combination therapy.

## Effects of radiation on the immune system

Traditionally, it is believed that radiotherapy leads to the death of tumor cells through irreversible damage to DNA. Many studies have found that the local killing effects of radiotherapy can be enhanced or reduced by stimulating or inhibiting the immune response in two different ways (58–60). Radiotherapy is involved in the modulation of many immune processes, such as cancer antigens release and presentation, T lymphocytes priming and activation, T cells recruitment and accumulation into tumor, T lymphocytes recognition and killing of tumor cells (61). The regimens of radiotherapy and the biological characteristics of the tumor also affect changes in immune responses.

## *In situ* tumor vaccine induced by radiation

Radiation results in the release of DNA DSBs from tumor cells into the cytoplasm (62). Cytosolic DNA is sensed by the cyclic GMP-AMP synthase stimulator (cGAS–STING) pathway of interferon genes. cGAS is a pattern recognition receptor that triggers the production of interferon I (IFN-I) through the downstream linker stimulator of interferon genes (STING) (63–65). IFN-I can stimulate dendritic cells (DCs) and T cell activation. This is critical for converting tumors into *in situ* vaccines (66). There is clinical evidence that IFN-I signaling is activated in spontaneously retreating tumors (67) and in metastases highly infiltrated by T cells (68, 69).

Radiation can induce immunogenic cell death (ICD), which can induce (local and/or systemic) release of tumor-associated antigens (TAAs), especially tumor neoantigens (TNAs) (70, 71). ICD is defined as a type of regulated cell death characterized by the release of damage-associated molecular patterns (DAMPs) after cells lose membrane integrity. DAMPs include calreticulin (CRT), the chromatin stabilization protein high-mobility group box 1 (HMGB1), adenosine triphosphate (ATP), and chaperons of the family of heat shock protein (e.g. HSP70) (72, 73). ICD leads to an adaptive immune response by favoring DC cross-presentation of tumor antigens to T cells. This can enhance anti-tumor immune responses and improve tumor control (72).

DAMPs and cytokines play important roles in radiation-induced ICD. First, calreticulin (CRT) is translocated from the endoplasmic reticulum to the cell surface and can act as an “eat-me” signal to antigen-presenting cells (APCs) (especially DCs and macrophages), *via* binding CD91 (a 2-macroglobulin receptor) (74, 75). This induces the subsequent release of cytokines, such as interleukin-6 (IL-6) and tumor necrosis factor alpha (TNF-α) (76). The CRT-CD91 interaction also mediates the recruitment of APCs to tumors, followed by DC phagocytosis of tumor cells and efficient presentation of tumor antigens to T cells. This ultimately leads to the activation of anti-tumor immune responses (77). Radiation can further enhance the endocytic activity of APCs by interfering with the CD47-signal regulatory protein α (SIRPα) phagocytic checkpoint pathway (78–81). CD47 is a marker of self-”don’t eat me signal”, and its loss on senescent or damaged cells leads to homeostatic phagocytosis (82). Importantly, CD47 is overexpressed in many tumors, and CD47 blockade has been identified as an attractive immunotherapeutic target (83, 84). Radiation induced loss of CD47 has been reported to enhance immune-mediated tumor clearance (78). Second, high-mobility group box 1 (HMGB1) is released from dying, necrotic, damaged tumor cells into the immune milieu and exerts robust immunomodulatory effects by binding to Toll-like receptor (TLR)-4 and TLR-9 (85, 86). HMGB1 can promote DCs maturation and migration to lymph nodes for antigen cross-presentation to naive T cells (87). Third, the release of ATP, which binds to the purinergic receptor P2X7, acts as a “find me” signal for monocytes and DCs, leading to the activation of NLRP3/ASC/caspase-1 inflammasome, and ultimately induce the production of IL-18 and IL-1β (88). IL-1β promotes the activation of IFN-γ-producing tumor antigen-specific CD8^+^ T cells (89). Fourth, HSP70 can be translocated from the cytoplasm to the extracellular matrix under conditions of radiation-induced cellular stress (90). HSP70 can activate monocytes, macrophages, and DCs by binding to CD14, CD40, CD91, Lox1 and Toll-like receptors (TLR2 and TLR4) (91). These results showed that the release of danger signals is critical for activating of antigen-presenting cells and for enhancing the immune response to tumor cells.

The cumulative effects of these molecular signals promote DCs phagocytosis of tumor cells, thereby facilitating DCs processing of tumor-derived antigens and subsequent DC-mediated cross-presentation to CD8^+^ cytotoxic T lymphocytes to release or induce type I interferons. Overall, radiation can induce ICD, an important pathway for activating antitumor immunity, which can transform tumors into an “*in situ* vaccine”.

## Abscopal effect induced by radiation

In 1953, the abscopal effect was first described as the regression of unirradiated tumors in a patient receiving radiation therapy (92). Over past decades, the abscopal effect is of great interest among radiation oncologists, but it remains a rare and poorly understood phenomenon in the clinic. In the era of cancer immunotherapy, many studies have found that radiotherapy combined with immunotherapy can enhance the abscopal effect (93–95). The key mechanism of this abscopal effect is radiation-induced *in situ* vaccination through liberating TAAs (7, 96). These neoantigens are then taken up by APCs, which are involved in the cross-priming of naive CD8^+^ T cells. Activated tumor-specific CD8^+^ cytotoxic T cells can move to the primary tumor and the metastatic lesions, activate systemic immunogenicity, induce abscopal effects, and control the growth of irradiated and non-irradiated tumors (60, 97).

## Reprogramming the tumor microenvironment through radiation

The tumor microenvironment (TME) is the internal environment on which tumor survival and development depends, and is associated with tumor growth, progression, and metastasis (98, 99). The dynamic changes in the TME lead to tumor cell variant selection. This results in the complexity of cancer heterogeneity and influences responses to different therapeutic strategies (100–102). TME can be segregated into four immune phenotypes based on tumor mutational burden and the presence of an inflammatory gene signature enriched for IFN-γ response genes (103). Chen et al. (61) classified TME into three types: an immune-inflamed, an immune-deserted and an immune-excluded. The TME of inflamed type, a “hot” phenotype with highly infiltration of CD4^+^ and CD8^+^ T cells, is accompanied by myeloid cells and monocytic cells. In addition, the immune cells are located in proximity to the tumor cells. Excellent responses to anti-PD-L1/PD-1 agents are most often in patients with inflamed tumors (104–106). On the contrary, the TME of deserted type refers to a “cold” phenotype lacking T lymphocytes infiltration in either the parenchyma or the stroma of the tumor. These deserted tumors rarely respond to therapeutic PD-L1/PD-1 antibodies (104). The TME of the immune-excluded type is an intermediate state characterized by the presence of abundant immune cells. However, the immune cells do not penetrate the tumor parenchyma, but instead remain in the stroma surrounding tumor cell nests (107, 108). Clinical responses are uncommon after anti-PD-L1/PD-1 treatment of these immune-excluded tumors. There is evidence of stroma-associated T cell activation and proliferation, but no infiltration (109). It is not clear how radiotherapy induces an immune-activating TME and radiotherapy leads to an immunosuppressive TME.

## Immune-enhancing effects of radiation in TME

More and more evidences indicated that radiotherapy can enhance innate and adaptive immune responses to tumors, thereby enhancing tumor responsiveness to radiation (110–113). Radiation therapy can induce the *in situ* tumor vaccine, thereby promoting the activation and maturation of DCs. DCs take up TAAs from damaged tumor cells and move to draining lymph nodes, and then present TAAs to T cells. Activated T cells can move to tumors to kill tumor cells. In addition, radiotherapy can upregulate the NK pathway to mediate tumor cells killing.

The *in situ* vaccination effect of radiation contributes to the uptake, processing and presentation of TAAs by DCs (such as CD11c^+^CD11b^+^ APCs) (114, 115). DCs (specialized APCs) can cross-presenting extracellular antigens, especially cell-associated antigens, to CD8^+^ T cells (116, 117). Many studies have shown that radiation can increase the levels of tumor-associated DCs, enhance the mobilization of these cells into draining lymph nodes, augment DCs maturation, and promote the ability of DCs (59, 60, 118). CD40 agonists are known to enhance DC function by increasing the surface expression of major histocompatibility complex (MHC) molecules and the production of proinflammatory cytokines (119). The cross-priming process requires the cognate T-cell receptors (TCR) to recognize the peptide major histocompatibility complex (MHC), which requires the costimulatory molecules CD80/86-CD28/cytotoxic T lymphocyte-associated protein 4 (CTLA4) and CD40L-CD40. Radiation can upregulate MHC-I molecules on tumor cells, thereby enhancing TAA presentation (120). This enhances tumor cells recognized by cytotoxic T cells specific to tumor antigen, and lysis of tumor cells by cytotoxic T cells. Radiation induces an increase in MHC I antigen presentation through three different mechanisms: (1) a proteasome-dependent increase in cytosolic peptide levels; (2) activation of the mTOR pathway leads to increased translation of proteins; (3) an increased generation of radiation-specific peptides (120). In addition to these cell intrinsic mechanisms of MHC-I induction, radiation-induced IFN-γ induces MHC-I upregulation (121). Therefore, radiation can increase MHC-I levels in some tumors with low endogenous MHC-I, thereby increasing immune-mediated attack. Furthermore, radiation upregulates the NK pathway by activating natural killer group 2D (NKG2D) ligands, and increasing NK cell cytotoxicity, tumor infiltration, and the production of many cytokines (112, 122). In addition, radiation can upregulate Fas expression by tumor cells, resulting in increased cytotoxic T cell lysis through a Fas/FasL-dependent mechanism (123). Radiation can induce the expression of cytokines and chemokines, such as CXC-motif chemokine (CXCL) 9, CXCL10, CXCL11 and CXCL16, thereby promoting the recruitment of effector CD8 and T-helper 1 CD4 T cells (124, 125). Radiation induces increased expression of vascular cell adhesion molecule 1 (VCAM-1) and intercellular adhesion molecule 1 (ICAM1) in tumor vessels, thereby promoting tumor infiltration by T lymphocytes (7, 126). Many studies have indicated that the presence of tumor-infiltrating lymphocytes, especially effector T cells, before therapy is associated with better survival in cancer patients (127, 128). Anitei et al. found that the densities of CD3^+^ T cells and cytotoxic CD8^+^ T lymphocytes were significantly correlated with disease-free survival and overall survival in patients with rectal cancer treated with chemoradiotherapy (129). Therefore, radiation induces the release of chemokines that subsequently enrich the T−cell infiltrate, and enhance priming of infiltrating T cells, thereby providing a positive immunological outcome.

**Figure 1:**
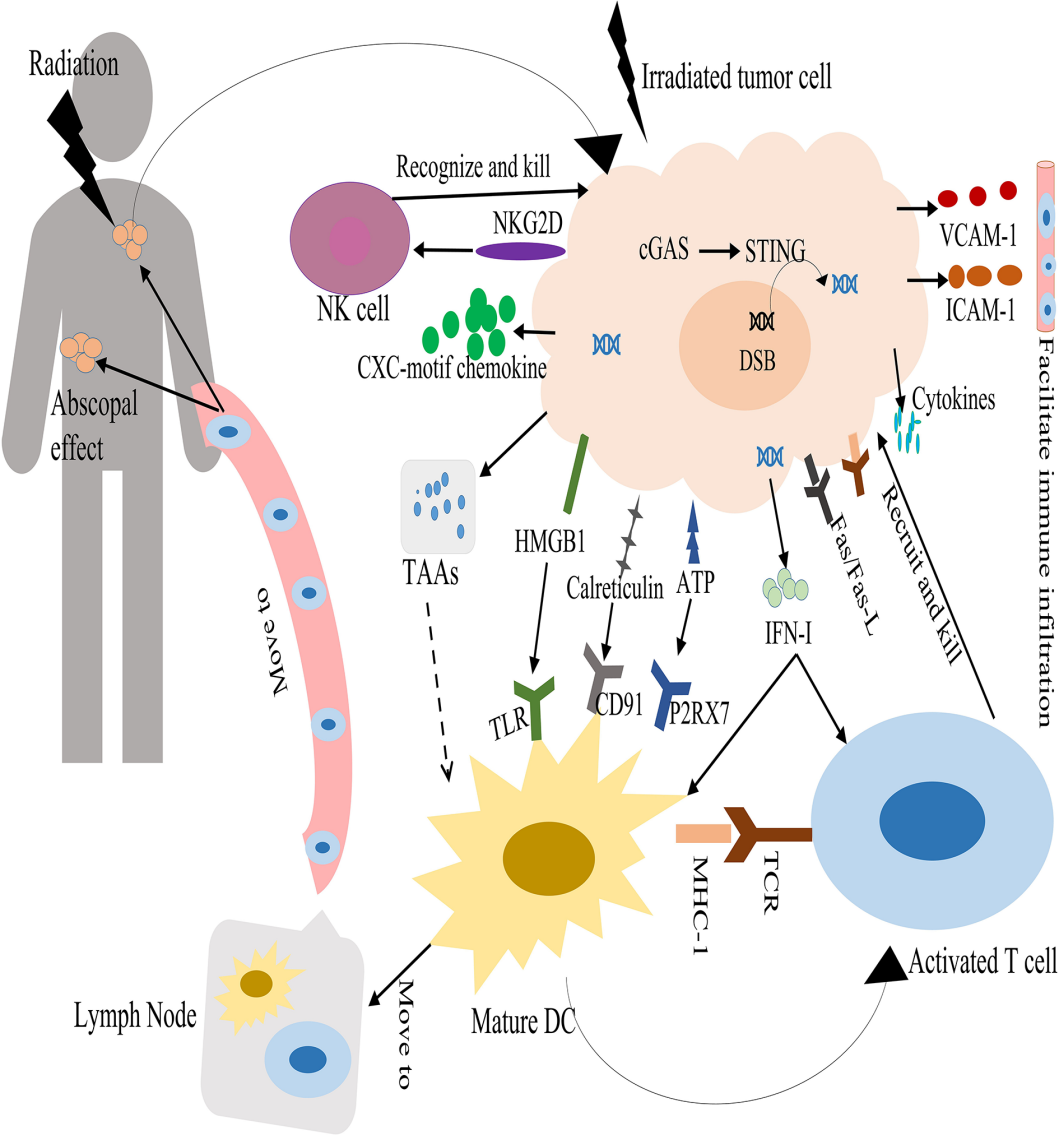
Antitumor immune enhancement of radiotherapy. Radiation therapy causes DNA DSBs in tumor cells and is sensed by the cGAS–STING pathway, resulting in the production of interferon I (IFN-I). In turn, IFN-I can stimulate dendritic cells (DCs) and T cell activation. Radiation therapy can induce immunogenic cell death. This releases danger-associated molecular patterns that promotes the activation and maturation of dendritic cells. DCs take up tumor-associated antigens (TAAs) from damaged tumor cells and travel to draining lymph nodes, and then present the TAAs on major histocompatibility complex class I (MHCI) to T cells through the T-cell receptor (TCR). Activated T cells move to the irradiated tumor and non-irradiated tumors through the blood circulation. Radiation can upregulate Fas and MHC-I molecules expression on tumors, and increase the release of cytokines and chemokines by tumor cells. This promotes the recruitment of activated T cells to kill tumor cells. In addition, radiation can upregulate the NK pathway to mediate tumor cells killing.

It is clearly that radiation can act on multiple tumor compartments to stimulate the tumor immune system. The antitumor immune-enhancing effects of radiotherapy were shown in **Figure 1**.

## Immunosuppressive effects of radiation in the TME

In addition to modulating the TME to generate antitumor immune responses, radiation can lead to immunosuppression of the TME and induce the expression of molecules that prevent DCs from cross-presenting tumor antigens and/or T cells to kill tumor cells. It is necessary to study this topic, because the suppression of the immune microenvironment leads to worse prognosis, and it may also be a legitimate therapeutic target.

Many studies have shown that radiation can lead to the recruitment of regulatory T cells (Tregs), myeloid derived suppressor cells (MDSCs) and tumor associated macrophages (TAMs) in the tumor microenvironment (123, 130, 131). Tregs are a subset of CD4^+^ T cells characterized by the expression of the transcription factor fork head box P3 (FOXP3). Tregs produce the cytokines transforming growth factor beta (TGF-β) and IL−10. This suppresses effector-T−cell activation and stimulates the suppressive functions of MDSCs (132). These results indicate that Tregs in tumors develop enhanced immunosuppressive properties after radiotherapy. Many studies indicate that the presence of highly suppressed Tregs in the circulation may represent a highly immunosuppressive environment induced by chemoradiotherapy, at least temporarily, in patients with glioblastoma and head and neck or cervical cancer (133–135). Therefore, targeting Tregs and/or their immunosuppressive effector molecules may be the key to reversing immunosuppression (136–138). After radiation therapy, the increased MDSCs can suppress the activation of both CD4^+^ and CD8^+^ T-cell responses in the TME *via* secretion of arginase-1 (ARG1) and nitric oxide synthase 2 (NOS2) (139, 140). In addition, MDSCs promote blood vessel formation and tumor regrowth (141, 142). Many studies in a variety of tumor models have shown that radiotherapy induces the recruitment of macrophages into tumor sites (123, 143). Radiation-induced recruitment of TAMs was dependent on increased expression of the chemokine CSF-1 (144). Although M1 macrophages can promote inflammation and antitumor immune responses, the M2 phenotype can promote tumor growth, angiogenesis, and metastasis after radiation (145, 146). Irradiated tumor cells release oxygen and nitrogen radicals that promote the polarization of macrophages from an inflammatory M1 phenotype into a tumor-supporting M2 phenotype. These M2 macrophages secrete the anti-inflammatory cytokines IL-10 and TGF-β, as well as the enzyme arginase-1, which lead to T cell suppression (147, 148). TGF-β can promote extracellular matrix production and angiogenesis, resulting in tumor cell proliferation, adhesion and metastasis (149, 150). TGF-β can impede anti-tumor immunity post-radiation by suppressing the effector functions of T-cells and natural killer cells, inhibiting DC maturation, promoting M2 macrophage polarity and the conversion of CD4 ^+^ T-cells into immunosuppressive Tregs (151). Radiation can stimulate the upregulation of immune checkpoint inhibitory molecules, such as programmed cell death ligand 1 (PD-L1) on tumor cells and PD-1 or CTLA-4 on cytotoxic T cells (CTLs). This can directly inhibit cytotoxic immune cell effector functions (152, 153). Therefore, when radiotherapy is combined with immune checkpoint inhibitors (such as anti-PD-1 antibody, anti-PD-L1 antibody and anti-CTLA4 antibody), T cell activity directed against tumor cells can be increased.

**Figure 2:**
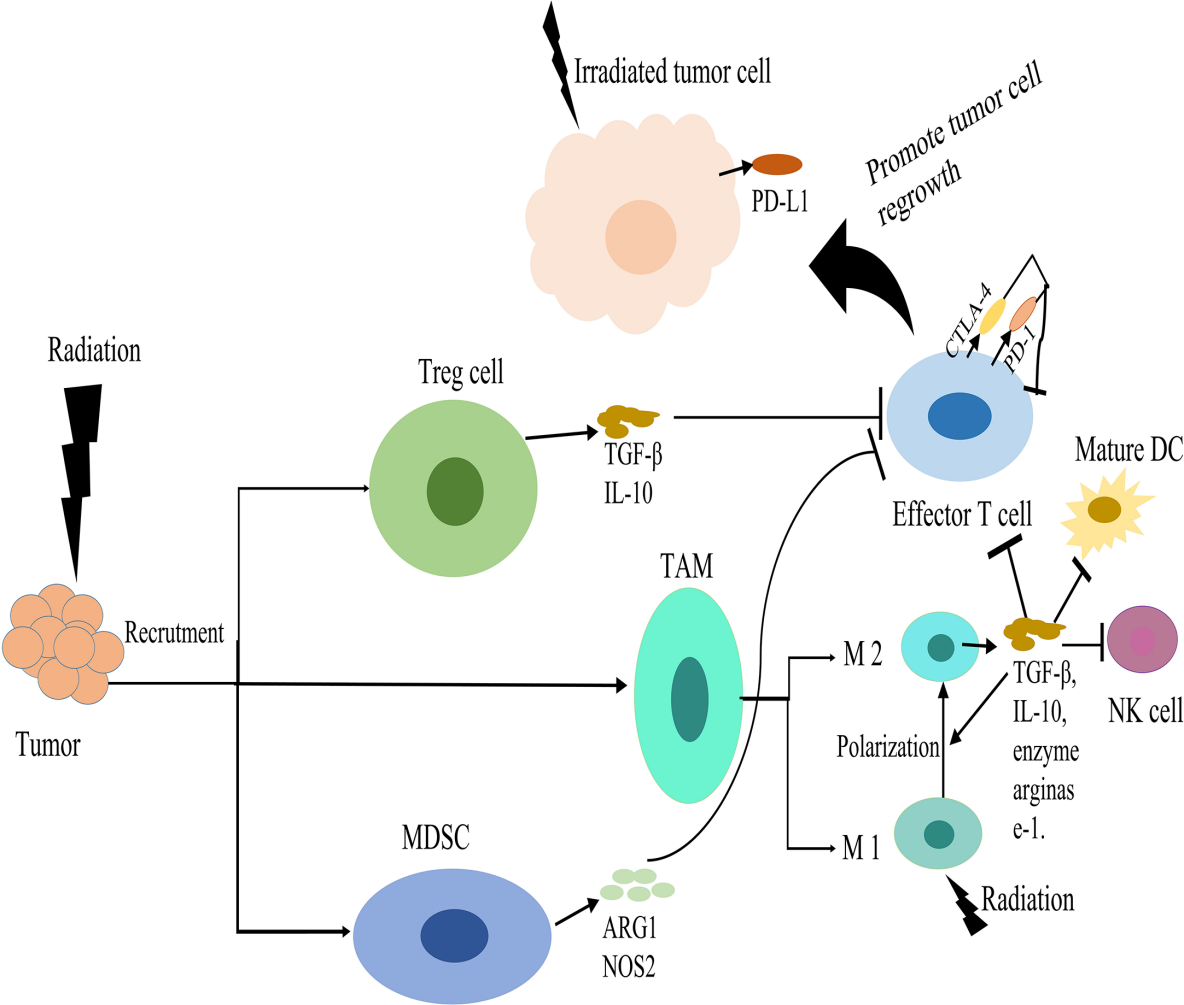
Immunosuppressive effects of radiotherapy. Radiation induces the recruitment of regulatory T cells (Tregs), myeloid derived suppressor cells (MDSCs) and tumor associated macrophages (TAMs) in the tumor microenvironment (TME). Tregs produce transforming growth factor beta (TGFβ) and IL-10, which suppress effector-T-cell activation. MDSCs can suppress the activation of T-cell responses *via* secretion of arginase-1 (ARG1) and nitric oxide synthase 2 (NOS2). Radiation can promote the polarization of macrophages from an inflammatory M1 phenotype into a tumor-supporting M2 phenotype. These M2 macrophages secrete IL-10 and TGFβ and the enzyme arginase-1, which suppress T cell. TGFβ can suppress the effector functions of T-cells and natural killer (NK) cells, inhibiting DC maturation, and promoting M2 macrophage polarity. Radiation stimulates upregulation of immune checkpoint inhibitory molecules, such as programmed cell death ligand 1 (PD-L1) on tumor cells and PD-1 or CTLA-4 on cytotoxic T cells, which down regulate T cell activation. These effects suppress the antitumor immunity and promote tumor cell regrowth.

In summary, radiation can promote the recruitment and activation of DCs and cytotoxic T cells through a variety of mechanisms, but this may be counteracted by the migration of suppressive immune cells. This presents an opportunity to combine radiation with immunomodulatory agents to improve tumor control. The immunosuppressive effects of radiotherapy were shown in **Figure 2**.

## The best combination of radiation therapy and immunotherapy

Radiation is increasingly used to control tumors locally, especially SBRT, with high rates of local control and significant benefits in terms of overall survival in many randomized trials (20, 154, 155). However, local tumor recurrence and distant tumor metastasis frequently occur when radiation therapy is used alone (156). Therefore, it is necessary to combine radiation therapy with other treatment options.

Immunotherapy has attracted great interest, and has become an established pillar of cancer therapeutics (99, 157). Unfortunately, most immunotherapeutic strategies are not effective in inducing tumor regression when used alone, and a large number of patients do not respond or become refractory to immunotherapy (106, 158, 159). The overall response rate to anti-CTLA-4 antibody is around 15%, while the response rate to PD-1/PD-L1 antibodies was < 25% (160). There are serval reasons to prevent immunotherapy from reaching its full potential. First, the priming of tumor antigen-reactive T cells is insufficient. Second, the infiltration of antitumor effectors into the tumor was weak. Third, the tumor microenvironment is highly immunosuppressive. Fourth, cancer cells effectively evade recognition by immune effectors with impaired tumor-associated antigen presentation (101, 161, 162). It is a great clinical challenge to overcome immunotherapy resistance.

Many studies showed that radiotherapy and immunotherapy are complementary. Irradiated tumors exhibit distinct patterns of immunogenicity, thereby improving response to immunotherapy (93). In addition, tumors with immunotherapy are more sensitive to radiotherapy. This can promote the localized treatment of tumors. Many studies found that radiation with ICIs can successfully treat metastatic cancers. This not only induced a local response, but also significantly regressed distant lesions outside the irradiation field (163–167). Theelen et al. pointed out that the addition of radiotherapy to pembrolizumab immunotherapy significantly improved abscopal responses and survival in patients with metastatic non-small-cell lung cancer (NSCLC), compared with pembrolizumab alone (29). This indicates that radiotherapy can convert non-responders to ICIs into responders.

Although radiotherapy combined with immunotherapy can improve the immune response, not every combination of radiation and immunization has been validated in clinical trials (32, 168). To further improve the anti-tumor ability, we need to select the appropriate patient population, explore the optimal radiotherapy regimens (dose, fractionation and volume), immunotherapy regimens (such as CTLA-4 inhibitors and PD-1/PD-L1 inhibitors), the sequence of radiotherapy and immunotherapy, and reduce the immunosuppressive effects and toxicity of radiotherapy.

## The appropriate radiotherapy regimens in combination with immunotherapy

Radiotherapy is a double-edged sword that is associated with immune activation and immune suppression. Therefore, it is necessary to study the optimal dose and fraction of radiotherapy to achieve optimal anti-tumor effects in combination with immunotherapy.

## High-dose radiotherapy promotes tumor immunogenicity

Many studies have shown that HDRT (such as SBRT) in combination with ICIs is more likely to cause tumor cell necrosis, enhance anti-tumor immunity, and lead to significant tumor control (94, 169). HDRT has showed a more potent immunogenic effect against cancer cells than conventional radiotherapy (usually 1.8–2 Gy per day) (23, 25). The conventional radiotherapy usually lasts for several weeks. Therefore, lymphocytes can be rapidly cleared from the irradiated field, reducing tumor antigen-specific T cell populations through sustained site-specific cytotoxicity. HDRT takes advantages over traditional radiation therapy when combined with immunotherapy.

Exposed tumor to a radiation dose ranging from 5 and 12 Gy per fraction, the number of infiltrated CD8^+^ cytotoxic T cells and NK cells were increased, while the number of Tregs was decreased. This is associated with the release of more anti-cancer cytokines, such as IFN-γ and TNF-α, and less immune suppressor cytokines, such as TGF-β and IL-10 (93, 170). Morisada et al. used hypo-fractioned radiation (8Gy*2f) or low-dose daily fractionated radiation (2Gy*10f) combined with anti-PD-1 antibody to treat mice bearing established syngeneic MOC1 oral carcinomas or MC38-CEA colon adenocarcinomas. They found that high-dose and low-dose fractionated radiation alone showed similar primary tumor control. However, anti-PD-1 antibody plus 8Gy*2f radiation rather than 2Gy*10f radiation, statistically significant enhanced CD8^+^ cell dependent primary and abscopal tumors control by inducing expression of IFN and IFN-responsive genes on tumor cells (171). Lan et al. compared ablative hypo-fractionated radiotherapy (AHFRT, 23Gy/2f/9d) versus conventional fractionated radiotherapy (36Gy/9f/9d) in mice bearing tumors from Lewis lung carcinoma or melanoma B16F10 cells, under the same conditions with biological equivalent dose (BED). They showed that AHFRT combined with anti-PD-L1 antibody presented a superior efficacy in controlling tumor growth and augmenting systemic anti-cancer immunity. The mechanism is that AHFRT suppressed the recruitment of MDSCs into tumors by regulating the VEGF/VEGFR axis, reduced MDSC-associated PD-L1 expression and increased the cytotoxicity of CD8^+^ T cells (25).

Many studies have shown that the abscopal effect was mainly observed in the combination of hypo-fractionated radiation regimens with ICIs. Dewan et al. used TSA and MCA38 cells to construct mouse tumor models. They found that fractionated radiation (8 Gy*3f or 6 Gy*5f) combined with anti-CTLA-4 antibody rather than single-dose radiation (20 Gy*1f) can induce an abscopal effect. In addition, 8 Gy*3f was more effective than 6 Gy*5f in eliciting systemic anti-tumor immunity combined with anti-CTLA-4 antibody (172). They further found that 20 Gy and 30 Gy single dose can attenuate cellular immunogenicity by inducing the DNA exonuclease Trex1 in various cancer cells, thereby degrading cytoplasmic DNA in irradiated cells (24). Cytosolic DNA stimulates secretion of IFN-β by cancer cells following activation of the DNA sensor cGAS and its downstream effector STING, which mediates optimal *in situ* vaccination (173). In fact, it was observed that the higher the dose per fraction, the more Trex1 was induced, resulting in significant DNA degradation. Therefore, the fractionated dose above the threshold (varies between 12 and 18 Gy in different cancer cells) for inducing Trex1 can result in downstream abrogation of IFN-β production, reducing DC recruitment and activation. Finally, it fails to induce systemic antitumor immune response (24). These results provide references for better selection of the radiotherapy regimens. However, these results also need to be validated clinically.

However, like conventional radiotherapy, HDRT can suppress tumor-reactive immunity by increasing the infiltration of Tregs and MDSCs, inducing an M2-like phenotype, and releasing TGF-β and IL-10 (143, 174). Lin et al. studied the effects of HDRT (8Gy/f) with and without anti-Gr-1 using syngeneic murine allograft prostate cancer models. They demonstrated that HDRT induced an early rise of MDSCs, followed by an increase of functionally active CD8 tumor-infiltrating lymphocytes. However, systemic depletion of MDSCs by anti-Gr-1 did not augment the antitumor immunity of HDRT because of the compensatory expansion of Treg-mediated immune suppression. This indicates that it is necessary to block MDSCs and Tregs for enhancing radiotherapy-induced antitumor immunity (33). Monjazeb et al. found that although HDRT induces an increase in CD8^+^ T cells and CD8^+^/PD1^+^/Ki67^+^ T-cells in the radiation field, HDRT may lead to a decrease in the ratio of M1/M2 macrophages in the tumor microenvironment (175). Furthermore, HDRT can inhibit the anti-tumor immune response by inducing tumor vascular damage. This can limit the infiltration of cytotoxic T lymphocytes into the tumor, and increase the hypoxic area (176, 177). Therefore, it is necessary to study the suppression of HDRT on the immune system, because it leads to poor prognosis, and it may be a reasonable therapeutic target.

In clinical practice, the combination of HDRT and immunotherapy is sometimes not superior to immunotherapy alone. Theelen et al. conducted a multicenter, randomized phase 2 study in advanced NSCLC patients who were treated with pembrolizumab either alone or after SBRT (8Gy*3f). They found that the overall response rate at 12 weeks was 18% in the pembrolizumab alone arm *vs* 36% in the pembrolizumab plus SBRT arm without statistical difference. In addition, no improvement in PFS or OS was achieved after the addition of SBRT (168). McBride et al. conducted another randomized clinical trial of nivolumab versus nivolumab plus SBRT (9 Gy*3f) in patients with metastatic or recurrent head and neck squamous cell carcinoma. The addition of SBRT to nivolumab did not statistically improve the objective response rate, OS or PFS, and there was no evidence of an abscopal effect in unselected patients (32). Therefore, ICIs and SBRT have synergistic local effects, but rare abscopal effects.

In conclusion, HDRT combined with immunotherapy does not always induce immune-enhancing antitumor effects and is only effective in a small subset of tumor patients. Tumor progression can still occur even if the complete remission is achieved. It is necessary to explore the best comprehensive treatment strategy.

## Low-dose radiotherapy reverses tumor-suppressing immune system

Although HDRT in combination with ICIs shows promising efficacy for clinical application, the treatment outcome still needs to be further optimized. A recent theory proposed that LDRT can modulate the TME, perhaps revolutionizing tumor treatment efficacy. LDRT usually refers to doses below a threshold, that is, the amount of doses less than that can physically damage DNA or kill cancer cells directly (178). The most common LDRT doses are 0.5-2 Gy/fraction, with total doses up to 1-10 Gy (179, 180). According to previous reports, LDRT modulated the immune suppressive stroma by downregulating TGF-β, repolarizing macrophages to favor the M1 over the M2 phenotype, and significantly enhancing the infiltration of effector CD4 T cells and NK cells. LDRT improved the efficacy of anti-PD1 and anti-CTLA4 agents, thereby promoting the overall systemic antitumor response (41). We will describe the ways that LDRT modulates the immune system in detail.

First, LDRT promotes the differentiation of macrophages to an M1-like phenotype. M2 macrophages can suppress antitumor immunity, and promote a radioresistant phenotype by secreting immunosuppressive mediators, such as IL-10 and TGF-β (181). Therefore, transforming the type of macrophages is critical to improve the immune enhancing effect. Felix Klug et al. (179) demonstrated that LDRT (0.5-2 Gy) can effectively transform M2 macrophages to iNOS^+^ M1 phenotypes, resulting in strong CD4^+^ and CD8^+^ T cells infiltration into human pancreatic carcinomas. After application of 0.5 Gy, the irradiated tumors contained the highest number of T cells, accompanied by an increase in CD4^+^ FoxP3^+^ T cells. In addition, LDRT can induce vascular normalization through crosstalk between macrophages and T cells. LDRT promoted T cell-mediated tumor eradication and prolonged survival (179). Prakash et al. irradiated advanced tumor-bearing Rip1-Tag5 mice with LDRT (2Gy*2f). They found profound changes in the inflammatory tumor microenvironment, characterized by induction of M1-related effecter cytokines as well as reduced cytokines of tumor-promoting and M2-related effecter cytokines (182). Furthermore, LDRT can program macrophages differentiation to an M1-like phenotype by ameliorating the hypoxia problem of tumors. Tumor hypoxia is known to be performed by angiogenesis-promoting HIF-1. This promotes angiogenesis, thereby interfering with tumor infiltration of CD8^+^ T cells and retuning of M1 phenotypic macrophages across the inert endothelium. Finally, it results in immunosuppressive effects (183–185). Nadella et al. demonstrated that LDRT (2 Gy) can downregulate HIF-1 in irradiated tumors, thereby supporting the differentiation of naive macrophages toward the M1 phenotype (186). Therefore, solving the hypoxia problem of bulk tumors can enhance the immune efficacy. LDRT has also been clinically observed to promote the differentiation of M1-type macrophages (175). Monjazeb et al. conducted a multicenter phase 2 study of 20 patients with histologically confirmed metastatic microsatellite-stable colorectal adenocarcinoma who had received at least one line of chemotherapy. These patients were randomly assigned to repeated LDRT or HDRT with PD-L1/CTLA-4 inhibition. They found that LDRT has the potential to increase the ratio of M1/M2 macrophages (175).

Second, LDRT can promote anti-tumor cytotoxicity of NK cells. LDRT can augment the direct expansion and cytotoxicity of NK cells through the P38-MAPK pathway (187). In addition, Sonn et al. found that when purified NK cells were irradiated with 0.2 Gy, the toxicity of NK cells was enhanced, while cell proliferation and apoptosis were unaffected (188). Cheda et al. (189) compared BALB/c mice that received or did not receive LDRT (single dose of 0.1 or 0.2 Gy), which were then injected with sarcoma cells. They found that the number of pulmonary tumor colonies was significantly reduced, and the cytolytic function of NK cells was significantly stimulated in the irradiated mice compared with the control group. In addition, NK-inhibitory anti-asialo GM1 antibody can totally abolish the tumor suppressive effect of LDRT. These results indicate that LDRT suppresses the development of experimental tumor metastases by stimulating the cytolytic function of NK cells.

Third, LDRT enhances T-cell infiltration. Herrera et al. (190) reported that LDRT of murine tumors promoted T-cell infiltration and responded to combinatorial immunotherapy in an IFN dependent manner. The mechanism is that LDRT induces CD4^+^ cells with characteristics of exhausted effector cytotoxic cells. One subset expressed NKG2D and exhibited proliferative capacity, as well as a unique subset of activated DCs expressed the NKG2D ligand RAE1. Zhou et al. established an *in vivo* lung cancer model. They found that LDRT activated T cells and NK cells, and promoted splenocyte cytotoxicity and T cell infiltration in the tumor tissues (191). Hashimoto et al. found that low-dose total body irradiation (0.2 Gy) increased the proportion of CD8^+^ cells in splenocytes, and even tumor-infiltrating lymphocytes were predominantly CD8^+^. Low-dose total body irradiation (0.2 Gy) inhibited lung and lymph node metastasis in tumor-bearing rats (192). In addition, low-dose total body irradiation of 2 Gy represents a powerful tool to foster CD4^+^ T cell-based cancer immunotherapies by favoring T helper 1 cells driven antitumoral immunity (193).

Fourth, LDRT can affect the function and activity of regulatory T cells (Tregs), thereby enhancing antitumor immunity. Tregs belong to a group of T lymphocytes that possess a negative immune regulatory function. The increased numbers of these cells in liver, breast, and ovarian cancer are closely related to the immune escape, occurrence, and development of tumor cells. Wang et al. found that LDRT (total 0.45 Gy) of the spleen can shrink tumors and increase the survival rate of rats with liver cancer. The mechanism by which LDRT enhances the immune effects may be that LDRT reduces the ratio of CD4^+^CD25^+^Treg/CD4^+^ in the blood and Foxp3^+^, IL-10, TGF-β and cytotoxic T lymphocyte-associated antigen 4(CTLA-4) expression (194). Liu et al. found that LDRT significantly reduced the percentage and absolute numbers of CD4^+^CD25^+^Foxp3^+^ regulatory T cells in naive mice, whereas CD4^+^CD44^+^/CD8^+^CD44^+^ effector memory T cells were greatly increased in naive mice (195). These results indicate that LDRT is a potential approach to overcome the tumor immunosuppressive microenvironment.

Finally, LDRT enhances the efficacy of ICIs. Barsoumian et al. (41) established mouse tumor models and irradiated the tumors with different doses. They found that LDRT alone (1Gy*2f) can effectively prolong survival by controlling tumor growth. The anti-tumor efficacy was further significantly enhanced when combined with anti-PD1 and anti-CTLA-4 drugs. This may be because LDRT can significantly activate CD4 and CD8 T cells, and enhance NK cell infiltration and M1 macrophage polarization and reduce TGF-β cytokine. Nowosielska et al. (196) found that LDRT to the whole-body (0.1 or 1.0 Gy) combined with anti-CTLA-4 antibody and anti-PD-1 antibody and NVP-AUY922 significantly reduced tumorigenesis in mice, and inhibited the clonogenic potential of Lewis lung cancer cells *in vitro*. By using targeted radionuclide therapy to semi-selectively deliver radiation to mouse tumors, Patel et al. found that low-dose targeted radionuclide therapy enhances the response of immunologically “cold” tumors to ICIs. After the combination of targeted radionuclide therapy and ICIs, 45-66% of mice exhibited complete responses and tumor-specific T-cell memory, while only 0% with targeted radionuclide therapy or ICIs alone. The reason is that the combination therapy activates the production of proinflammatory cytokines in the TME, promotes tumor infiltration and clonal expansion of effector CD8^+^ T cells, and reduces spontaneous metastasis (197). Furthermore, the addition of LDRT to PD-L1/CTLA-4 blockade was feasible and safe in clinical practice (175). In a phase I clinical study, Herrera et al. found that the adding LDRT (0.5 or 1 Gy per fraction) to the combination immunotherapy group showed a therapeutic effect for an overall disease control rate of 87.5% in patients with immune desert tumors. In addition, using a single-sample gene set enrichment analysis approach, they observed that responding tumors exhibited an increase in Th1, CD8^+^ and T_EM_ signatures, whereas non-responding tumors exhibited an upregulation of M2 macrophage and neutrophil signatures (190). However, in some cases, LDRT combined with immunotherapy failed to induce effective antitumor immunity. Schoenfeld et al. (198) conducted an open-label, multicenter, randomised, phase 2 trial involving 90 patients with metastatic NSCLC resistant to PD(L)-1 therapy. Patients were randomly assigned to 3 arms, durvalumab plus tremelimumab alone, or in combination with LDRT (2 Gy/4f), or in combination with HDRT (24 Gy/3f). Radiotherapy was delivered at 1 week after initial durvalumab–tremelimumab administration. They found that neither HDRT nor LDRT increased the response to combined PD-L1 plus CTLA-4 inhibition.

The rationale for using LDRT is not necessarily to ablate or kill the tumors, but to activate the immune system to eliminate these lesions in concert with other therapeutic approaches. Clinically, LDRT has the following advantages over HDRT. First, the toxicity of LDRT is low. If radiotherapy is to be delivered simultaneously to several lesions within an organ, it is difficult to meet the dose limit to the organ at risk with SBRT, whereas dose limits will be easier to meet with LDRT. Therefore, LDRT is dosimetrically more feasible than SBRT in the treatment of large tumor volumes, or even a whole organ. Second, LDRT is safer for patients who have received radiation in the past. There is a minimal concern about exceeding normal tissue dose-constraints when re-radiation is performed by LDRT. Finally, LDRT is easier to be delivered. In clinical practice, LDRT can be performed through three-dimensional technology, while HDRT requires specialized imaging, respiratory gating, and even gold fiducials implantation.

**Figure 3:**
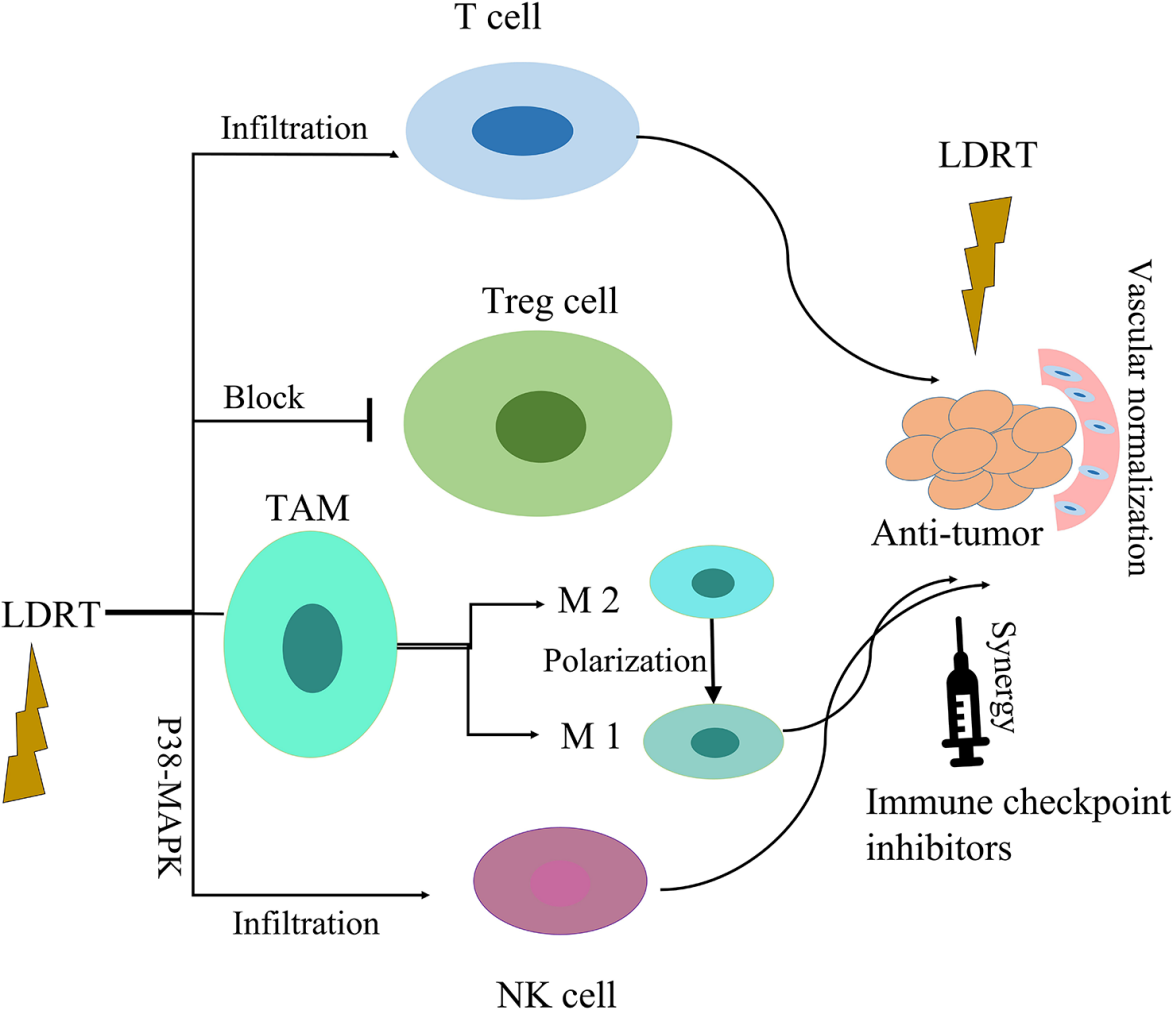
Immunomodulatory effects of LDRT in tumor microenvironment. LDRT modulates the tumor microenvironment by repolarizing macrophages to favor the M1 over the M2 phenotype, blocking regulatory T cells, and enhancing the infiltration of effector CD4 T cells and NK cells. Low-dose radiation can improve the efficacy of immune checkpoint inhibitors.

In sum, LDRT provides an emerging approach to address limitations of radioimmunity mechanisms. It is necessary to further study this important method Immunomodulatory effects of LDRT in tumor microenvironment are shown in **Figure 3**.

## High-dose and low-dose radiotherapy synergistically enhance antitumor immune responses

HDRT and LDRT work differently on the immune system. We take full advantage of the advantages of HDRT and LDRT to enhance anti-tumor immune responses. HDRT can increase the release and presentation of tumor antigen, and stimulate immune cell activation. However, LDRT can modulate the TME to stimulate immune cell infiltration into the tumor stroma and the tumor bed of distant tumors. Next, we will introduce the preclinical and clinical studies of HDRT combined with LDRT, i.e., multimodal radiotherapy.

Studies showed that HDRT in combination with LDRT was superior to HDRT or LDRT alone in tumor control and activation of anti-tumor immunity (39, 199). Savage et al. (39) designed a novel radiation scheme (PAM-RT), a single high-dose radiation (22Gy) followed by post-ablation modulation with four daily low-dose fractions (0.5Gyx4f). They found that PAM-RT localized to the primary tumor in 3LL tumor-bearing mice can significantly delay tumor growth and increase survival. They treat metastatic breast cancer (4T1) mice with PAM-RT, where the primary tumor received high-dose irradiation (20 Gyx3f) and metastatic organs received low-dose irradiation (whole lung, 0.5 Gyx4f). Survival was significantly increased after whole-lung radiated by low dose compared with primary tumor ablative radiotherapy alone. The mechanism is that PAM-RT can promote remodeling of the TME in the primary tumor as well as the metastatic site by reducing Tregs, activating macrophages to an inflammatory phenotype, and promoting infiltration of CD8^+^ CTLs into metastatic tumors. Liu et al. (199) used a combination of hypo-fractionated radiation therapy (8Gy×3f) targeted primary tumor with low-dose total body irradiation (0.1Gy) in a syngeneic mouse model of breast or colon carcinoma. They found that low-dose total body irradiation alone did not delay the growth of primary or secondary tumors. Hypo-fractionated radiation therapy led to a significant growth delay of the irradiated primary tumors, but did not have a systemic immune related response to secondary tumors. However, the combination of low-dose total body irradiation and hypo-fractionated radiation therapy significantly delayed the growth of both the primary and secondary tumors, and translated into the best OS with systemic antitumor response characteristics. The mechanism is that the combination therapy (HDRT and LDRT) induced infiltration of CD8^+^ T cells, IFN-γ^+^ CD8^+^ T cells and DCs in unirradiated tumors, reduced granulocytic-myeloid-derived suppressor cells and M2 macrophages, and increased the percentage of antitumor eosinophil population. These results indicate that LDRT can serve as a potential therapeutic agent for patients with metastatic cancer. Their therapeutic potential is significantly enhanced when combined with HDRT.

Compared with the combination of ICIs with either LDRT or HDRT alone, the combination of LDRT and HDRT further enhanced the response to ICIs, resulting in an enhanced antitumor response (40–42, 197). Barsoumian et al. proposed the use of high dose and low dose radiation (RadScopal technique) with immune oncology agents (anti-TIGIT and anti-PD1 monoclonal antibodies) to against highly metastatic lung adenocarcinoma tumors in 129Sv/Ev mice. They found that the triple therapy can prolong the survival of treated mice, and halt the growth of primary and secondary tumors,and reduce the percentages of TIGIT^+^ exhausted T-cells and TIGIT^+^ regulatory T-cells (40). Yin et al. (42) compared HDRT/anti-PD1, HDRT/LDRT, or LDRT/anti-PD1 double treatments. They demonstrated that the enhancement of the abscopal response was achieved by triple therapy consisting of HDRT (8 Gy*3f) to treat the primary tumor, LDRT (2 Gy*1f) to treat the abscopal tumor, and anti-PD1 therapy. The enhanced abscopal effect was associated with increased infiltration of CD8^+^ effector T cells and upregulated expression of T cell-attracting chemokines. The triple treatment also improved the tumor response in patients with metastatic NSCLC and was well tolerated. In addition, HDRT combined with LDRT and double agent checkpoint blockers can effectively control metastatic tumors by increasing CD4^+^ effector T cells, enhancing NK cell activation, and increasing M1 macrophages in secondary tumors. Further clinical studies have shown that when the tumor burden was high, it was necessary to use HDRT to priming T cells at the primary tumor site, and LDRT to modulating the stroma of secondary (metastatic) tumors (41).

Surprisingly, the treatments combining HDRT with LDRT and immunotherapy have also achieved promising clinical results. Analyzing 26 cancer patients received LDRT (1-20 Gy in total), Menon et al. found that this was because of the scatter of HDRT or the intentional treatment at a second isocenter of LDRT. These patients underwent prospective clinical trials on the combination of radiotherapy and immunotherapy. They compared lesions that received LDRT with without radiation (< 1 Gy). 85% of lesions that received LDRT achieved PR/CR, while 18% of lesions that received no-dose (P=0.0001). This indicates that LDRT can increase systemic response rates of metastatic disease treated with HDRT and immunotherapy (43). They also conducted a phase II trial of ipilimumab with concurrent or sequential SBRT (50 Gy/4f or 60 Gy/10f) for metastatic lesions in the liver or lung. Some non-targeted lesions received LDRT (5–10 Gy) because they were anatomically close to another irradiated site. Further analysis showed that lesions that received LDRT were more likely to respond than those that did not (31% *vs* 5%, P=0.0091) (200). Patel et al. analyzed a phase II trial of HDRT (3–12.5 Gy/f up to 20–70 Gy total) with or without LDRT (1-10 Gy total; 0.5-2 Gy/f) for patients who had the metastatic disease that progressed on immunotherapy within 6 months. A total of 74 patients with NSCLC or melanoma were enrolled in the study. 39 patients received HDRT and 35 patients received the combination of HDRT and LDRT. There was no significant difference regarding disease control rate. However, the overall response rate for HDRT + LDRT *vs*. HDRT cohorts, lesions treated by LDRT was significantly improved rates of lesion-specific responses compared with nonirradiated lesions. This is because LDRT induced a remarkable increase of T- and NK cell infiltration into the irradiated lesions (180). These clinical studies indicate that HDRT and LDRT combined with immunotherapy can synergistically generate tumor-specific immune responses, thereby enhancing systemic antitumor effects.

In summary, multimodal radiotherapy, a combination of HDRT to stimulate T cell priming together with LDRT to modulate inhibitory tumor microenvironment, can enable immune cells to infiltrate into tumor bed and trigger antitumor responses. This provides a new treatment alternative for patients with advanced cancer after multiple lines of therapy, and brings immunotherapy into a new field of systemic disease control. Many clinical trials are investigating the efficacy and safety of different combinations of HDRT and LDRT in patients with advanced tumors (**Table 1**).

**Table 1:** Clinical trials of multimodal radiotherapy (HDRT and LDRT) in advanced tumors.

Trial number	Phase		Cancer type	Treatment strategy	Primary end points
NCT02710253	II	Single group	Hematopoietic and lymphoid cell neoplasm / Metastatic malignant solid neoplasm	SBRT(4f, 5f, or 10f) or EBRT(4f, 5f, or 10f) for any site of metastatic disease.	Disease control rate. Objective response. Incidence of adverse events.
NCT02416609	Not applicable	Single group	Advanced Pancreatic Cancer	In each cycle, Gem-based doublets will be administered concurrent with LDRT for a total of 4 cycles. If there is no progress, SBRT will be performed sequentially.	Progression free survival
NCT03085719	II	Not applicable	Head and neck cancer	Arm 1 is that HDRT (3f) is combined with pembrolizumab. Arm 2 is HDRT and LDRT in combination with pembrolizumab.	Overall response rate
NCT03812549	I	Single group	Stage IV NSCLC	SBRT (30Gy/3f) is first delivered to lung, in combination with LDRT starting from the 2nd day of SBRT, followed by sintilimab monotherapy starting within 7 days after the completion of radiotherapy. Sintilimab will be administered at 200mg every 3 weeks.	Safety and tolerability

## Challenges in clinical practice

Patient response to immunotherapy or immunotherapy combined with HDRT can be enhanced by delivering LDRT to certain tumor sites for the purpose of immunomodulating the TME, thereby promoting systemic propagation of anti-tumor immunity and the destruction of tumor by immune effector cells. However, it is necessary to further study clinical practices of high-dose and low-dose radiotherapy. For example, which tumor site should be treated with HDRT or LDRT? Whether HDRT and LDRT should be delivered to the same tumor site? What is the best sequence of HDRT and LDRT?

## Selection of irradiation sites for high and low dose radiotherapy

**Figure 4:**
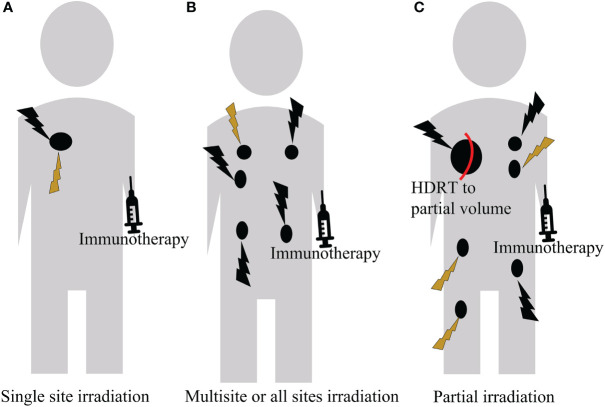
A personalized treatment regimen based on the patient’s performance status, clinical symptom, extent of tumor burden, and the immune type of the tumor microenvironment. **(A)** For a single tumor, HDRT can initiate T cell priming, followed by LDRT to modulate the tumor microenvironment. **(B)** For oligometastatic disease, HDRT can be delivered to all lesions. If it is infeasible and intolerable, it can be supplemented with LDRT. **(C)** For extensive metastatic disease, partial volume HDRT can be delivered to one or a few lesions, followed by LDRT to other lesions.

There are few literatures on the targeting volume selection in studies combing immunotherapy with multimodal radiotherapy (HDRT and LDRT). Based on existing reports and clinical experiences, we developed individualized strategies based on performance status of patients, clinical symptoms, extent of tumor burden, and the immune type of the tumor microenvironment (**Figure 4**). This individualized treatment regimen can not only control the tumor, but also improve the patient’s quality of life.

## Single site irradiation

For patients with only a single lesion, HDRT can be used to ablate the tumor for achieving the radical cure. This not only shrinks the tumor. but also promotes the release of antigens. Subsequent application of LDRT to modulate the tumor microenvironment can attenuate the immunosuppressive effects of SBRT, and increase immune effector cell infiltration, thereby synergistically enhancing the response to ICIs. However, it is necessary to study it in the future. At present, only a preclinical study has reported that HDRT followed by LDRT to the primary tumor can delay tumor growth and prolong survival in mice (39).

## Multisite or all sites irradiation

Tumors are heterogeneous (201, 202). This indicates that tumor associated antigens (TAAs) present in some tumor sites may be different from those in other tumor sites, or may not be equally immunogenic. Targeting a single metastatic site in patients with multiple metastases cannot unmask TAAs in another site unless those TAAs are shared. Therefore, useful antitumor immune responses are not activated systemically.

Monjazeb et al. (175) did not observe objective responses outside the irradiation field. This indicates that irradiating 1-2 lesions in combination with the immune checkpoint blockade is not sufficient to mediate systemic antitumor immunity in patients with refractory colorectal cancer. In addition, in the multicenter, randomized phase 2 study, 76 patients with recurrent metastatic NSCLC were randomized to either pembrolizumab alone or pembrolizumab to a single tumor site after SBRT (3 doses of 8 Gy). The overall response rate of the added SBRT group was twice that of the control group. However, the results did not meet the criteria of a meaningful clinical benefit endpoint (168). Luke et al. conducted a phase I study enrolling patients with metastatic solid tumors who had progressed on standard treatment. 69 patients were treated with SBRT (total of 30-50Gy/3-5f) and at least one cycle of pembrolizumab. SBRT was delivered to two to four metastases, but not all metastases were irradiated. They found that multi-site SBRT can limit the progression of existing metastases, and enhance anti-tumor immune responses. This improves outcomes in metastatic patients treated with pembrolizumab (203). Iyengar et al. (21) found that the addition of SABR to all severe disease sites significantly improved PFS without increasing toxic effects. In addition, studies have reported that multisite SBRT followed by pembrolizumab was safely tolerated (27, 204). These clinical experiences indicate that irradiation of multiple sites or all metastatic lesions can maximize systemic synergy.

Patients classified as having oligo-metastases may be candidates for HDRT delivery to all lesions for immune priming and local control. In addition, direct delivery of SBRT to all tumor sites ensures tumor sterilization. However, irradiation of all lesions by HDRT may sometimes be infeasible and intolerable in clinical practice because of dose volume constraints in normal tissue surrounding the tumors. Therefore, some tumor lesions can be supplemented with low-dose irradiation.

In sum, multiple target irradiation tends to induce stronger antitumor immunity and generate more frequent abscopal responses than a single target. Therefore, it is necessary to further study this approach in clinical settings.

## Partial irradiation

Systemic therapy is the standard of care in patients with non-oligometastatic cancers. However, the addition of SBRT can not only shrink the local tumor to induce an effect of *in situ* vaccination, but also relieve local symptoms, such as pain, obstruction and bleeding, etc. To enhance the systemic efficacy of immunotherapy, it is necessary to use HDRT to stimulate immune priming for the bulky or “cold” tumors. The partial irradiation may be considered in this case because of toxicity.

Preclinical experiments have shown that high-dose partial irradiation can delay tumor growth through immune activation. Markovsky et al. (205) treated 50% or 100% of tumors with radiation in a 67NR Murine Orthotopic Mammary Tumor Model and the less immunogenic Lewis Lung Carcinoma mouse model. They found that partial irradiation in immunocompetent mice resulted in a tumor response similar to full irradiation. This is because of the CD8^+^ T cell-mediated immune stimulation mechanism. Furthermore, a significant abscopal effect was elicited after hemi-irradiation of the primary tumor with a single dose of 10Gy in the 67NR model. Yasmin-Karim et al. (206) found irradiating a field smaller than the entire tumor volume showed the same or better distal effect than irradiating the entire tumor volume field, and significantly reducing healthy tissue damage. This is due to higher infiltration of cytotoxic CD8+ T lymphocytes in treated and untreated tumors.

The partial irradiation combined with immunotherapy is clinically feasible. Luke et al. found that partial irradiation by SBRT was performed when the metastases were greater than 65 mL. They compared patients with at least one tumor partially irradiated to those with fully irradiated tumors, and found no statistically significant tumor control rate at 3 months (27). They further found no statistically significant difference in objective response rate, PFS, and OS between patients who received full and partial SBRT at multiple sites in the presence of pembrolizumab. Furthermore, a clinical response at the irradiated site can be induced without irradiating the entire metastases (203). Lemons et al. (207) reported that partially irradiated tumors exhibited similar control as completely irradiated tumors in patients with metastatic solid tumors treated with pembrolizumab and SBRT. These indicate that partial volume SBRT is enough to activate immune priming.

A novel partial irradiation technique, spatially fractionated radiation therapy (SFRT), can also induce an antitumor immune response. SFRT can deliver a high dose to a large irradiation field that is segmented into several small units with steep dose gradients, which lead to reduce the normal tissue toxicity (208–211). In the study of Johnsrud et al. (212), whole tumor irradiation or SFRT (a single dose of 20 Gy) alone or in combination with ICI were tested in mice using a triple negative breast tumor. In the group of SFRT, they observed the abscopal immune response in contralateral tumors with obviously increased infiltration of both antigen-presenting cells and activated T cells, followed by an increase in systemic IFNγ production and ultimately a delay in tumor growth. Further studies are needed to explore the new partial irradiation technique.

In addition to immunotherapy for those patients with extensive metastases, it is beneficial to apply partial volume HDRT to one or several lesions to induce immunogenic cell death. Then, applying LDRT to other lesions for tumor microenvironment modulation can enhance abscopal effects, thereby reducing tumor burden.

## The optimal sequence of high and low dose radiotherapy

HDRT and LDRT combined with immune checkpoint blockades can improve local and systematic antitumor responses in advanced tumors. However, the optimal sequence of these therapies for optimal efficacy remains unclear.

For combination therapy, LDRT can be applied before or after HDRT. Many existing studies about the sequencing issue have different results. Savage et al. (39) divided C57BL/6 mice with palpable subcutaneous 3LL tumors into five treatment groups: no treatment, 24 Gy on day 1 or 5, four fractions of 1Gy followed by 20 Gy or 20 Gy followed by four fractions of 1Gy, four of which were treated with radiation therapy to the primary tumor. They found that 1Gyx4f after ablation radiation (20 Gy) showed the best tumor control and the longest survival. However, pre-treatment with low-dose radiotherapy resulted in minimal tumor control compared with single-dose ablative radiotherapy. This is due to the rapid growth of 3LL tumors, and ablative radiation targeting the larger tumors on day 5. This can affect the efficacy of the priming radiation. Therefore, it is necessary to control rapidly growing tumors with high-dose radiotherapy first, and then modulate the immune microenvironment with low-dose radiotherapy. However, other scholars found that sequential administration of LDRT followed by HDRT achieved superior antitumor immunity than the start of HDRT before LDRT. Liu et al. (199) administered HDRT to the primary tumor at 48, 72, 96, and 120 h after low-dose total body irradiation (L-TBI) in mouse tumor models. Starting HDRT at 72 h after L-TBI can achieve the best overall survival and the maximum abscopal effect. In addition, they compared the time of L-TBI 3 days before or after HDRT, or simultaneously with HDRT. The results showed that HDRT 3 days after L-TBI exhibited the best therapeutic effect, for example, a significant inhibition of tumor growth and improved survival of the treated mice. They found that L-TBI followed by HDRT can induce an adaptive immune response and protect the immune system of the mice. Zhou et al. observed that LDRT pretreatment before HDRT was able to ameliorate the HDRT-induced immune impairment and enhance the antitumor immunity (191). Therefore, it is necessary to conduct large preclinical and clinical trials for the optimal sequencing of HDRT and LDRT.

However, the optimal timing of the addition of immunotherapy to HDRT and LDRT remains unclear. Some scholars pointed out that immunotherapy is more effective after radiotherapy than before. This is because radiotherapy can promote the release of TAAs and destroy any pre-existing immune tolerance in the tumor periphery. Wei et al. (213) demonstrated that the administration of αPD-1 antibody after local tumor irradiation could induce a potent abscopal response while the addition of αPD-1 before radiation abrogated the abscopal effect. This antitumor efficacy was associated with the expansion of polyfunctional intratumoral CD8^+^ T cells, reduction of intratumoral dysfunctional CD8^+^ T cells, and expansion of reprogrammable CD8^+^ T cells. Many studies showed that the concurrent combination of anti-PD-L1 antibody and radiation achieved better tumor control than the sequential schedule (214, 215). Bestvina et al. conducted a randomized phase 1 trial comparing the combination of nivolumab and ipilimumab with sequential or concurrent multisite SBRT in patients with stage IV NSCLC. They found that the median PFS were 18.6 and 13.2 months for concurrent and sequential therapy, respectively. The concurrent treatment strategy was not more toxic than the sequential one (216). However, there are some different views. A phase 1 trial compared combined pembrolizumab with SBRT administered either prior to the first pembrolizumab cycle (arm A) or prior to the third pembrolizumab cycle (arm B). Their results indicated that ORR of arm B was significantly better than that of arm A (44.4% *vs* 0%) (217). This is because the administration of immunotherapy prior to SBRT stimulates antigen-presenting cells and effector T cells, thereby making these cells ready to respond to the tumor antigen efflux generated by SBRT (169). Therefore, it is necessary to further study the optimized sequence of high and low dose radiotherapy combined with immunotherapy according to the biological characteristics of tumors, the selection of immunotherapy drugs, and the effects of radiotherapy on the immune system.

## Conclusion

In this paper, we introduced a multimodal radiotherapy regimen (HDRT combined with LDRT) to synergistically enhance the local and systemic antitumor immunity, and improve the response to immunotherapy, thereby achieving the best anti-tumor effects. HDRT induces *in situ* tumor vaccine and primes cytotoxic T cells. LDRT modulates the tumor microenvironment, which in turn promotes the infiltration and lethality of immunocompetent cells. This multimodal radiotherapy regimen can be applied to primary tumor and metastatic lesions, thereby improving the local and systemic antitumor immunity. It is even possible to irradiate the whole organ with LDRT to boost immunity for widespread organ metastases, such as the lungs or liver. In clinical practice, it is possible to individually implement multimodal radiotherapy coupled with immunotherapy according to the patient’s performance status of patients, disease burden, and tumor immune microenvironment phenotypes. It is necessary to conduct a further study to solve those issues.

## Author contributions

XS: Conceptualization. XJ, JW, SL, HH, WJ and GC searched the literature. XJ and WJ wrote the first draft. XS, WD and BZ wrote and edited overall. All authors contributed to the article and approved the submitted version.
